# Plasma concentration of selected biochemical markers of endothelial dysfunction in women with various severity of chronic venous insufficiency (CVI)—A pilot study

**DOI:** 10.1371/journal.pone.0191902

**Published:** 2018-01-29

**Authors:** Magdalena Budzyń, Maria Iskra, Wojciech Turkiewicz, Zbigniew Krasiński, Bogna Gryszczyńska, Magdalena Paulina Kasprzak

**Affiliations:** 1 Department of General Chemistry, Chair of Chemistry and Clinical Biochemistry, Poznan University of Medical Sciences, Poznań, Poland; 2 Department of General Surgery, Medical Center HCP, Poznań, Poland; 3 Department of General and Vascular Surgery, Poznan University of Medical Sciences, Poznań, Poland; Centro Cardiologico Monzino, ITALY

## Abstract

**Background:**

Although the endothelial dysfunction is considered to be implicated in the pathogenesis of chronic venous insufficiency (CVI) the endothelial status in patients with venous disorders is still not fully evaluated. Therefore the aim of the study was to measure the concentration of selected markers of endothelial dysfunction: von Willebrand factor (vWf), soluble P-selectin (sP-selectin), soluble thrombomodulin (sTM) and soluble VE-cadherin (sVE-cadherin) in CVI women who constitute the most numerous group of patients suffering from venous disease.

**Materials and methods:**

Forty four women with CVI were involved in the study and divided into subgroups based on CEAP classification. Concentration of vWf, sP-selectin, sTM and sVE-cadherin were measured and compared with those obtained in 25 healthy age and sex-matched women.

**Results:**

It was found that the concentration of sTM increased and sVEcadherin decreased along with disease severity in CVI women. A significant rise of sTM was observed especially in CVI women, with the highest inflammation status reflected by hsCRP or elastase concentration, and in CVI women with a high oxidative stress manifested by an increased plasma MDA. A significant fall of circulating sVE-cadherin was reported in CVI women with moderate to highest intensity of inflammation and oxidative stress. There was no change in vWF and sP-selectin concentration at any stage of CVI severity.

**Conclusions:**

The results of the present study demonstrate the presence of endothelial dysfunction in women suffering from CVI which seems to progress with the disease severity and may be associated with inflammation and enhanced oxidative stress.

## Introduction

Chronic venous insufficiency (CVI) is a condition widely prevalent in Western societies. Globally, CVI may affect more than 60% of the adult population [1], and its high costs, both at individual and societal levels, have been well documented [2]. The most common manifestation of chronic venous insufficiency are telangiectases, reticular veins, and varicose veins. More severe symptoms include soft tissue edema, dermatitis, hyperpigmentation, lipodermatosclerosis and ulceration The etiology and pathophysiology of CVI have been intensively studied in the past decades. Various epidemiological studies agree that women tend to suffer from CVI more frequently than men [3,4,5,6]. They experience varicose veins, the most common manifestation of CVI, three to four times more often than men [7]. Due to the high prevalence of venous diseases among women they are adequate subjects to be included in the study of mechanisms and the risk factors of CVI development.

One known hypothesis assumes that the inflammation plays a central role in the pathogenesis of CVI [8,9,10]. The inflammatory mechanisms accompanied by the liberation of cytokines, proteases and reactive oxygen species (ROS) contribute to endothelial damage and further pathological remodeling of the vein wall [11]. Endothelial cells undergoing injury release a variety of soluble particles known as biochemical markers of endothelial damage or dysfunction (ED) [12]. Their evaluation is a simple and non-invasive method of endothelial function measurement, therefore they are quantified and investigated in conditions associated with an increased vascular risk, such as peripheral and coronary atherosclerosis, aortic aneurysm, diabetes mellitus, or rheumatoid arthritis [13,14,15,16,17]. It is well known that non-invasive endothelial function assessment has predictive value for the occurrence of cardiovascular event in primary and secondary prevention settings [18,19]. Moreover the association of improvement in ED with risk reduction for future cardiovascular events has also been demonstrated [18,20]. At present flow-mediated vasodilatation (FMD) has become the most widely used non-invasive technique to measure endothelial function. This technique measures the ability of arteries to respond with endothelial NO release during reactive hyperemia after a 5-minute occlusion of the brachial artery with a blood pressure cuff. Although the principle of this technique seems to be simple, its application is technically challenging and requires an extensive training and standardization [21,22,23,24]. Moreover, in some situations, the degree of reactive hyperemia may vary even under the same stimulus. Also, changes in the structure of blood vessels and impaired dilation may be limiting factors during FMD assessment [22]. Therefore, a simpler and more reproducible parameter of ED is still being investigated and biochemical markers seem to be an adequate candidate. The advantages of using of serum biomarkers in the assessment of endothelial status are the simplicity of the procedure and the fact that venous blood samples are widely used in laboratory routine [22]. Moreover, biochemical markers demonstrate an excellent sensitivity in reflecting ED, comparable with biophysical measurements [25,26,27]. It is also highly probable that liberation of biochemical markers into circulation by injured endothelial cells may precede the alterations in their function measured by biophysical techniques, such as FMD. The use of these biochemical markers in the prognosis and/or diagnosis of vascular disease is most common at the initial stages, but this is still a study area with a great potential.

The fact that CVI may be another type of pathology associated with ED is confirmed by histological examination revealing alteration in morphology of endothelial cells and disruption in endothelial layer integrity [18]. However, the number of studies evaluating the markers of endothelial status and their potential impact on CVI diagnosis or prognosis is still limited [28,29,30,31]. There is also lack of data indicating the factors which may potentially participate in ED in CVI development. Therefore, researches conducted on ED and their markers may give not only insights into pathophysiology of CVI, but also possible clinical opportunity to detect early disease, prevent severe complications such as venous leg ulcers, and assess response to treatments.

The aim of this pilot study was to evaluate selected biochemical markers of ED, namely von Willebrand factor (vWf), soluble P-selectin (sP-selectin), soluble thrombomodulin (sTM) and soluble vascular endothelial cadherin (sVE-cadherin) in the blood of women affected by CVI. The association of markers of ED with disease severity, demographical data (BMI value, age) and parameters of inflammation and oxidative stress (hsCRP, elastase and MDA concentration) was also analyzed to find their potential contribution to endothelial pathology in CVI.

## Materials and methods

### Patients

The group of patients consisted of 44 women, aged 26–65 years (mean age: 45±11) with primary varicose vein (VV), who underwent lower extremity VV excision. Preoperative, lower-extremity venous color duplex ultrasound scanning was performed on all patients and both the superficial and the deep venous systems were studied. Venous reflux was defined as flow in the inverted direction for a period longer than 0.5 seconds. Partial and complete venous obstruction was assessed by the degree of compressibility of the venous walls, with normal defined as complete compressibility. Superficial venous functional disease (SFD) and deep venous functional disease (DFD) were defined as reflux on ultrasound or abnormal compression in superficial and deep veins, respectively. In all cases the patency of the deep vein system as well as the lack of thrombotic changes were confirmed. The patients with peripheral arterial occlusive disease (ankle-brachial index < 0.9) or any other conditions that might result in leukocyte activation, such as diabetes, cancer, connective tissue disorders or infection within the previous six weeks were not included into the study.

All women had visible venous disease signs that corresponded to the Clinical Etiologic Anatomic Pathologic (CEAP) classification categories. There were 19 women with VV (class 2), 10 women with edema (class 3), 16 patients with pigmentation, lipodermatosclerosis, atrophie blanche, healed or active ulceration (class 4, 5 and 6). Therefore, the disease severity was the criterion for categorizing the women into two subgroups: with moderate CVI (included C2 and C3 classes) and severe CVI (included C4, C5 and C6 classes)

The control group consisted of 25 women, aged 36–60 (mean age: 42±7), all of whom were members of medical staff. They had no superficial functional disease (SFD) or deep functional disease (DFD) on ultrasound, no varicose veins, edema or trophic changes of the skin on physical exam and no reports of leg aching. The study procedure was approved by Bioethical Committee of the University of Medical Sciences in Poznan, and informed consent was obtained from all participants.

### Sample collection

Blood samples were drawn preoperatively from the arms of CVI women on the day of surgery, in the recumbent position after 10 minutes of rest. Samples were collected in EDTA tube with anticoagulant for plasma and serum tubes. After 30 minutes, the tubes were centrifuged at 3.000 rpm for 15 minutes. Serum and plasma samples were stored at temperature of -80°C until all of assays were performed. The study procedure was approved by the Bioethical Committee of the University of Medical Sciences in Poznan and informed consent was obtained from all participants.

### Laboratory analysis

The von Willebrand factor (vWf), soluble thrombomodulin (sTM), soluble P-selectin (sP-selectin), soluble VE-cadherin (sVE-cadherin), high sensitive C-reactive protein (hsCRP) and leukocyte elastase concentration were measured using enzyme-linked-immunosorbent assay (Abcam, UK; Gen-Probe Diaclone SAS, France; R&D System, USA; eBioscience, Austria; DRG International, USA; Hycult Biotech, The Netherlands). MDA concentration was measured using calorimetric assay kit (Abcam, UK).

### Statistical analysis

The statistical analysis were conducted using GraphPad Prism software 6.0 (GraphPad Software, San Diego, CA). The normality of quantitative variables were tested using the Kolmogorov-Smirnow or Shapiro-Wilk test. Any parameter not following the normal distribution was presented as a median and interquartile ranges and analyzed using non-parametric Mann-Whitney test. Categorical data and proportions were compared using Chi-square or Fisher`s exact test, as appropriate. Normally distributed, continuous variables were presented as a mean and standard deviation and analyzed using the Student’s *t* test. Multiple group comparisons were performed by one-way analysis of variance or Kruskal-Wallis test, respectively. The Pearson or the Spearman correlation coefficient was used to test the strength of any association between different variables. In all cases, *P* value ≤ 0.05 was considered significant.

## Results

After categorizing patients into appropriate subgroups according to CEAP classification, women with severe CVI showed to be older and had a higher BMI value (Table 1).

**Table 1:** Markers of endothelial dysfunction, inflammation and oxidative stress in women with moderate and severe CVI.

Parameter	Control (*n* = 33)	Moderate CVI (*n* = 28)	Severe CVI (*n* = 16)	*p*-value
Age (years)	45.18±10.53	41.72±9.15	51.44±10.22	0.001^a^
BMI (kg/m^2^)	24.52±6.34	22.67±4.83	26.19±5.06	0.027^a^
hsCRP (mg/L)	1.00 (0.12–1.86)	0.98 (0.13–3.15)	1.15 (0.56–2.84)	NS^b^
Elastase (ng/mL)	41.50 (24.50–65.75)	51.75 (19.00–81.25)	59.00 (25–172)	NS^b^
MDA (μM)	2.41 (2.03–4.06)	3.93 (2.92–5.40)	4.41 (3.47–5.93)	0.001^b^
vWf (mU/mL)	808 (723–957)	890 (649–961)	805 (627–997)	NS^b^
sTM (ng/mL)	0.96 (0.86–1.63)	1.23 (1.09–1.69)	1.43 (1.10–2.25)	0.027^b^
P-selectin (ng/mL)	26.16 (11.45–45.82	19.64 (8–36.36)	21.27 (10.27–52.50)	NS^b^
sVE-cadherin (ng/mL)	39.30 (36.19–42.11)	30.34 (23.36–34.44)	31.56 (26.31–41.90)	0.002^b^

^a^ Results shown as mean± standard deviation, one-way ANOVA test was used for comparison.

^b^ Results shown as median and interquartile range, Kruskull-Wallis test was used for comparison.

NS—not statistically significant

**Fig 1:**
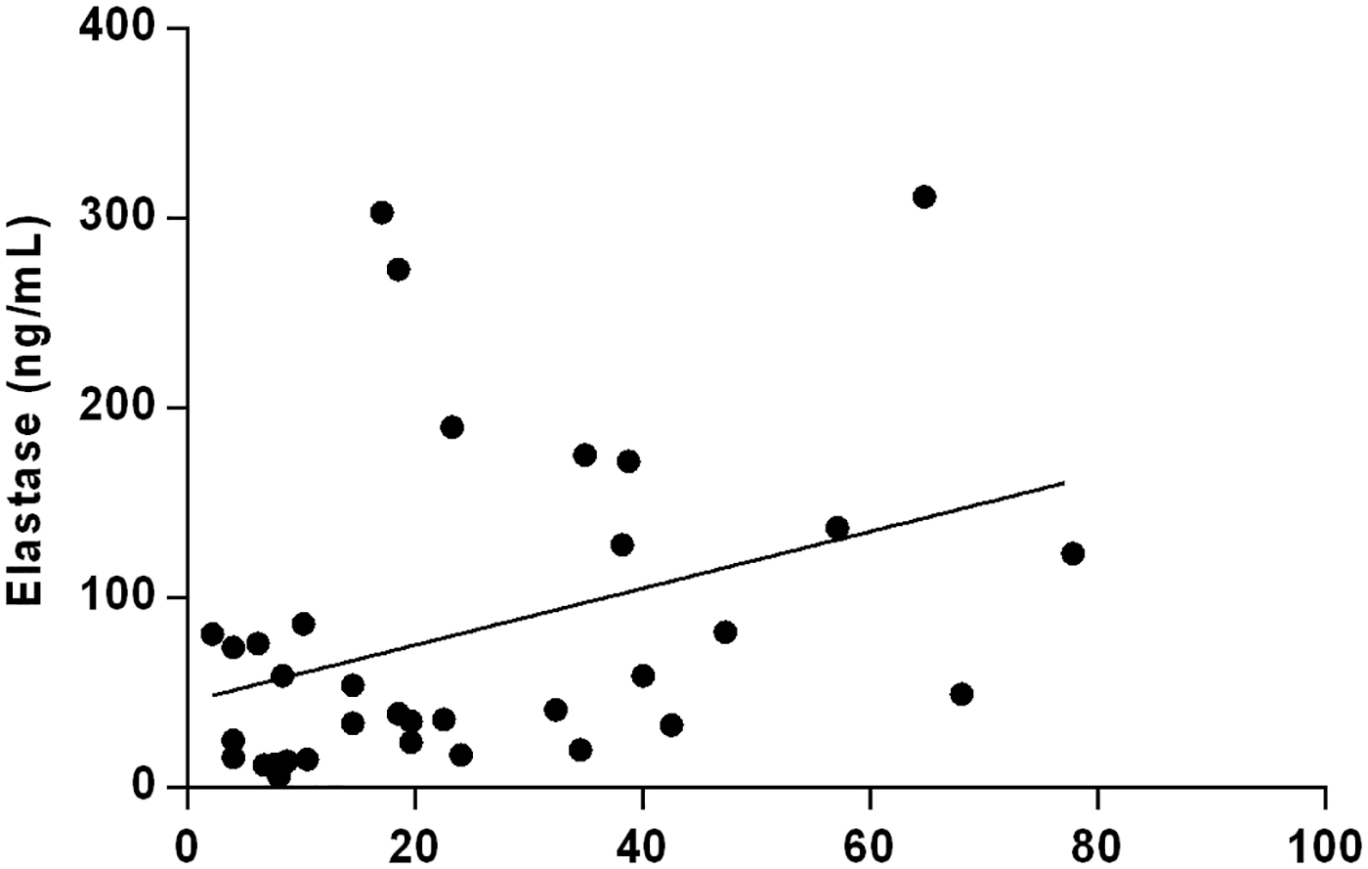
Correlation between sP-selectin and elastase. (Spearman correlation coefficient r = 0.419, *P* = 0.014).

**Fig 2:**
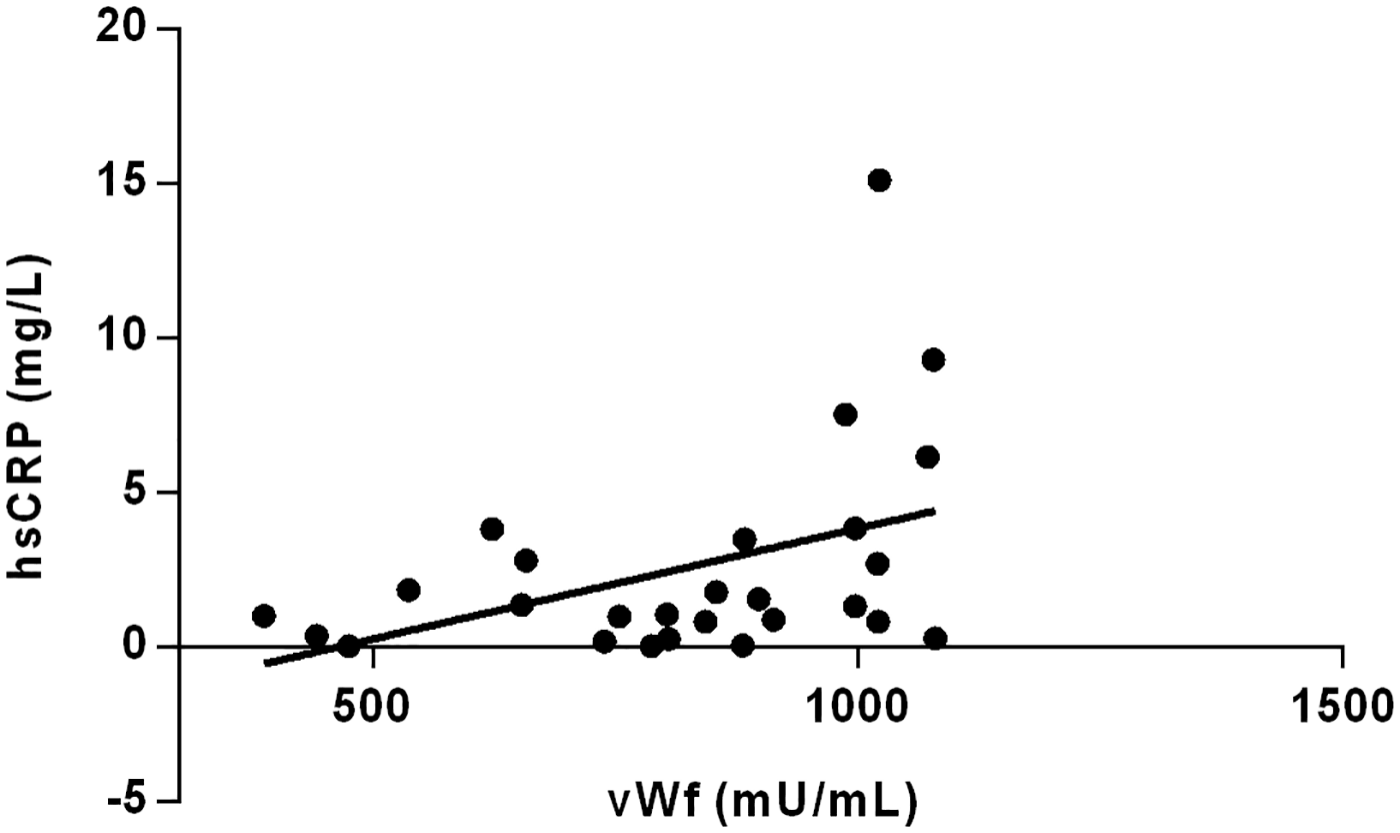
Correlation between vWF and hsCRP. (Spearman correlation coefficient r = 0.373, *P* = 0.049).

There was no difference in hsCRP and elastase concentration between studied subgroups and control (Table 1). It was demonstrated that MDA concentration tends to increase together with the disease severity (Table 1). The same trend was observed for sTM concentration which rose reaching the highest value in women with severe CVI (Table 1). The level of sVE-cadherin was decreased in women with moderate as well as severe symptoms of CVI. There was no difference in the concentration of vWf and sP-selectin between subgroups and control. Significant positive correlation between sP-selectin and elastase was found (r = 0.419, *P* = 0.014) in the whole group of CVI women (Fig 1). Moreover, an elevated vWf values correlated with an increased hsCRP concentration (r = 0.373, *P* = 0.049) (Fig 2).

To analyze the impact of inflammation and oxidative stress on endothelial function all examined CVI women have been divided into 3 groups using 25th and 75th percentiles of hsCRP, elastase and MDA concentration distribution, respectively, as cutoff points (group I—<25^th^ percentile, group II - 25^th^-75^th^ percentile, group III—>75^th^ percentile). Then the endothelial markers were compared between quartiles and control group.

**Fig 3:**
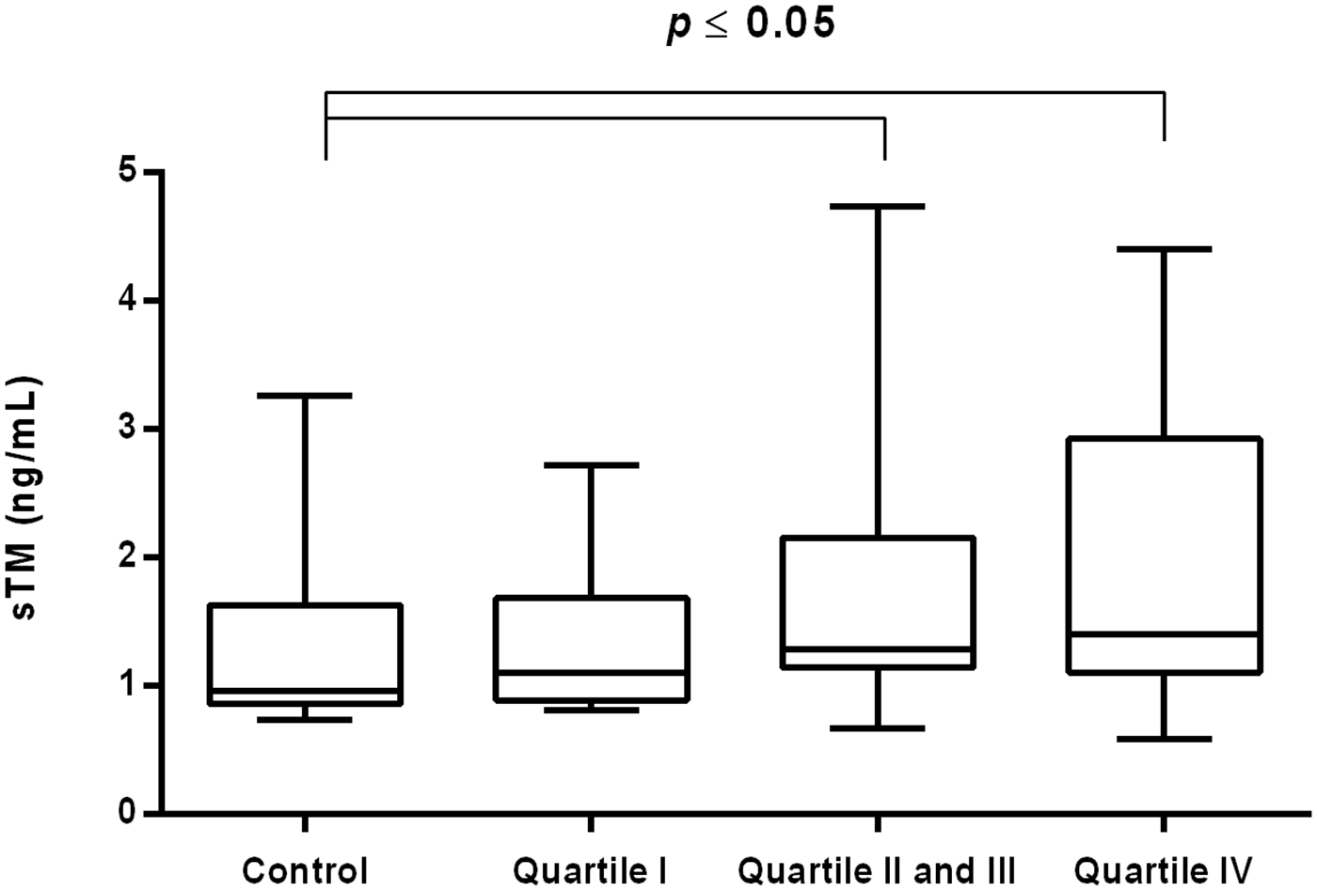
Concentration of sTM in CVI woman categorized into quartiles of plasma hsCRP. Box and whisker plots show median (central line), upper and lower quartiles (box) and range excluding outliers (whiskers). Data were analyzed using Kruskal-Wallis test followed by the Dunn`s multiple comparison test. * *P*≤0.05 was considered statistically significant.

The value of sTM was significantly increased in CVI women in the middle and upper quartiles of hsCRP in compared with control [1.23 (1.08–2.54) ng/mL vs. 0.96 (0.86–1.63) ng/mL *P* = 0.047; 1.40 (1.10–2.93) ng/mL vs. 0.96 (0.86–1.63) ng/mL *P* = 0.045] (Fig 3).

**Fig 4:**
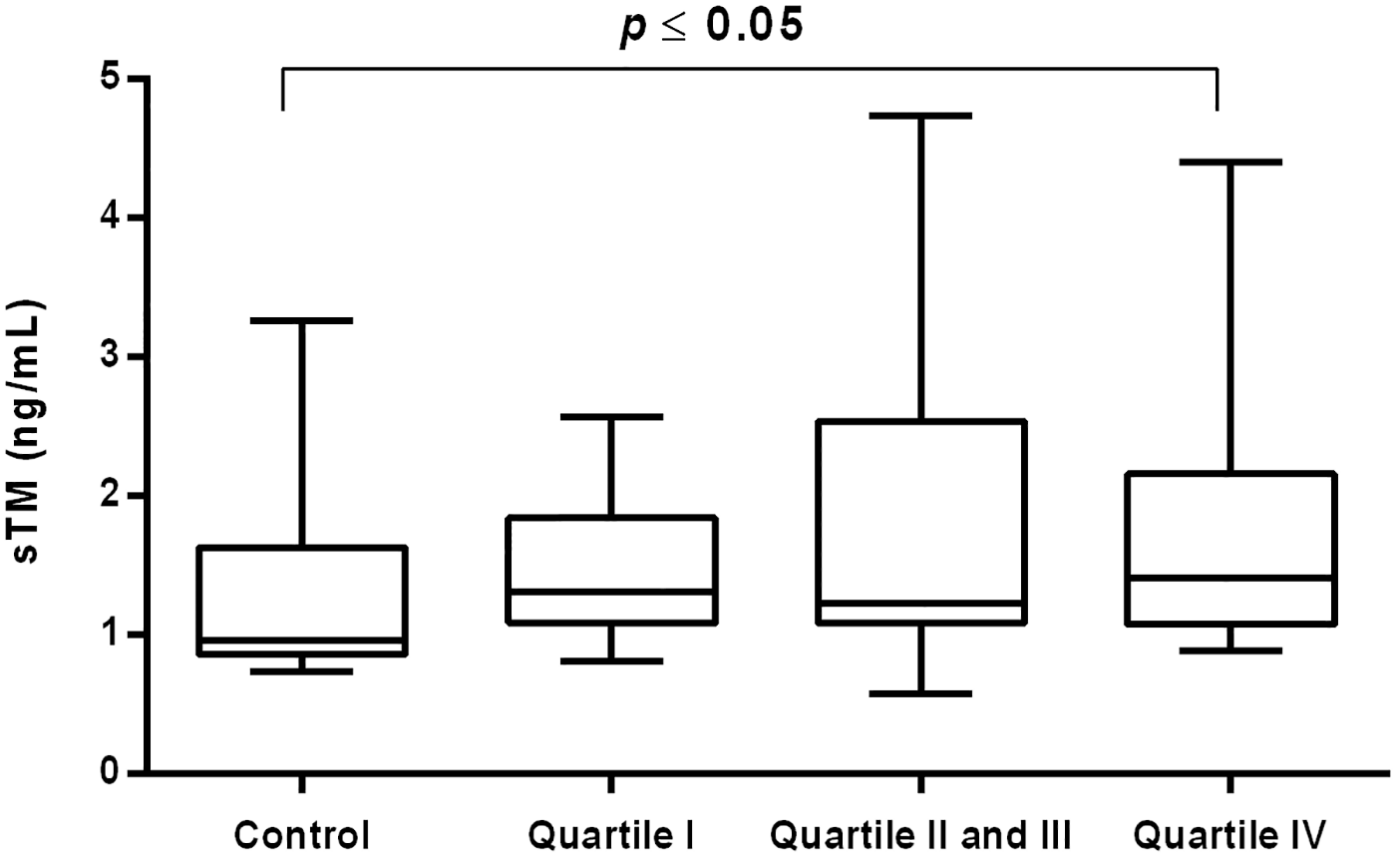
Concentration of sTM in CVI woman categorized into quartiles of plasma elastase. Box and whisker plots show median (central line), upper and lower quartiles (box) and range excluding outliers (whiskers). Data were analyzed using Kruskal-Wallis test followed by the Dunn`s multiple comparison test. * *p*≤0.05 was considered statistically significant.

**Fig 5:**
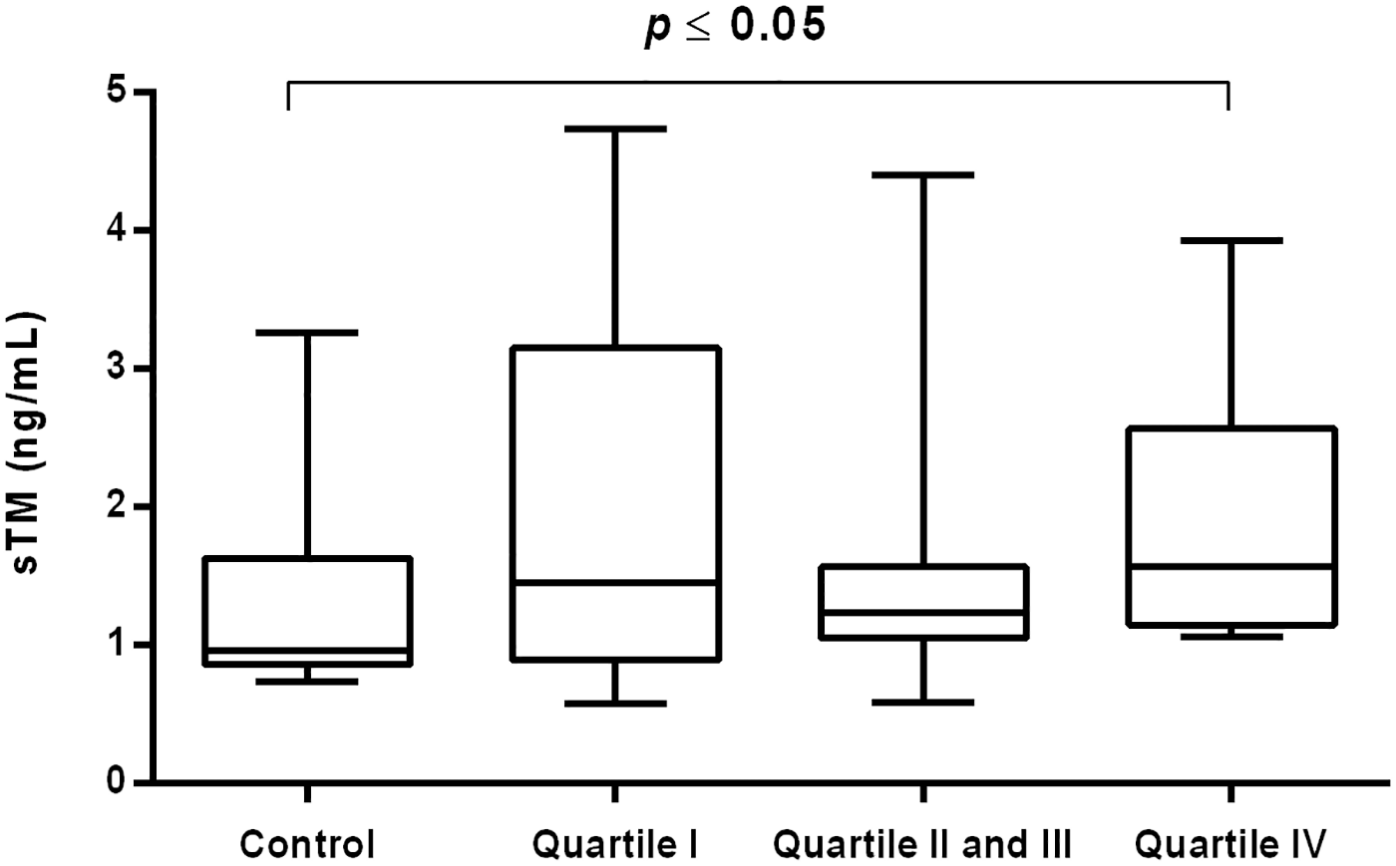
Concentration of sTM in CVI woman categorized into quartiles of plasma MDA. Box and whisker plots show median (central line), upper and lower quartiles (box) and range excluding outliers (whiskers). Data were analyzed using Kruskal-Wallis test followed by the Dunn`s multiple comparison test. * *p*≤0.05 was considered statistically significant.

The rise in sTM concentration was also noticed in CVI women in the highest quartile of elastase [1.41 (1.07–2.16) ng/mL vs 0.96 (0.86–1.63) ng/mL P = 0.049] and MDA concentrations [1.57 (1.15–2.57) ng/mL vs 0.96 (0.86–1.63) ng/mL *P* = 0.032], respectively (Figs 4 and 5).

**Fig 6:**
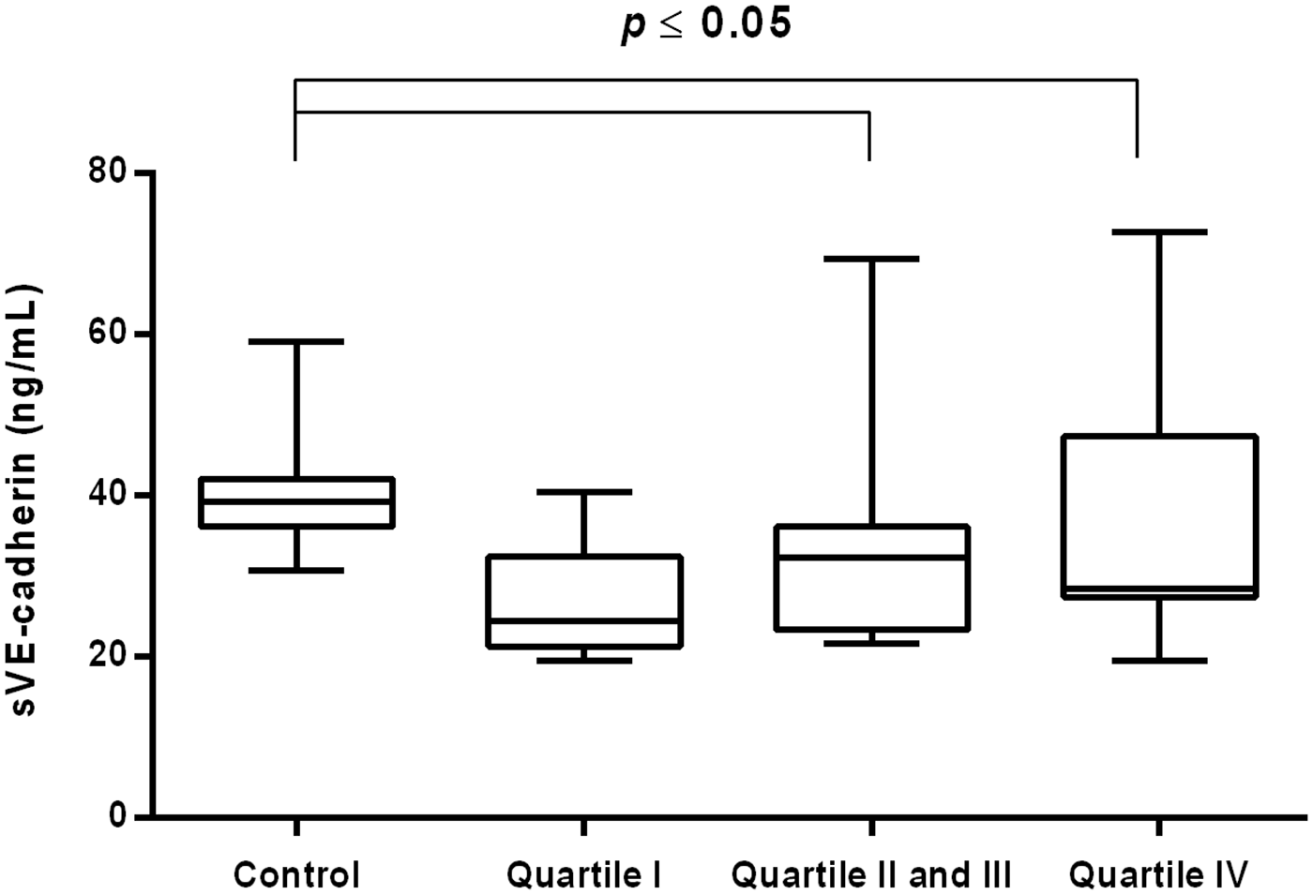
Concentration of sVE-cadherin in CVI woman categorized into quartiles of plasma hsCRP and control group. Box and whisker plots show median (central line), upper and lower quartiles (box) and range excluding outliers (whiskers). Data were analyzed using Kruskal-Wallis test followed by the Dunn`s multiple comparison test. * *p*≤0.05 was considered statistically significant.

**Fig 7:**
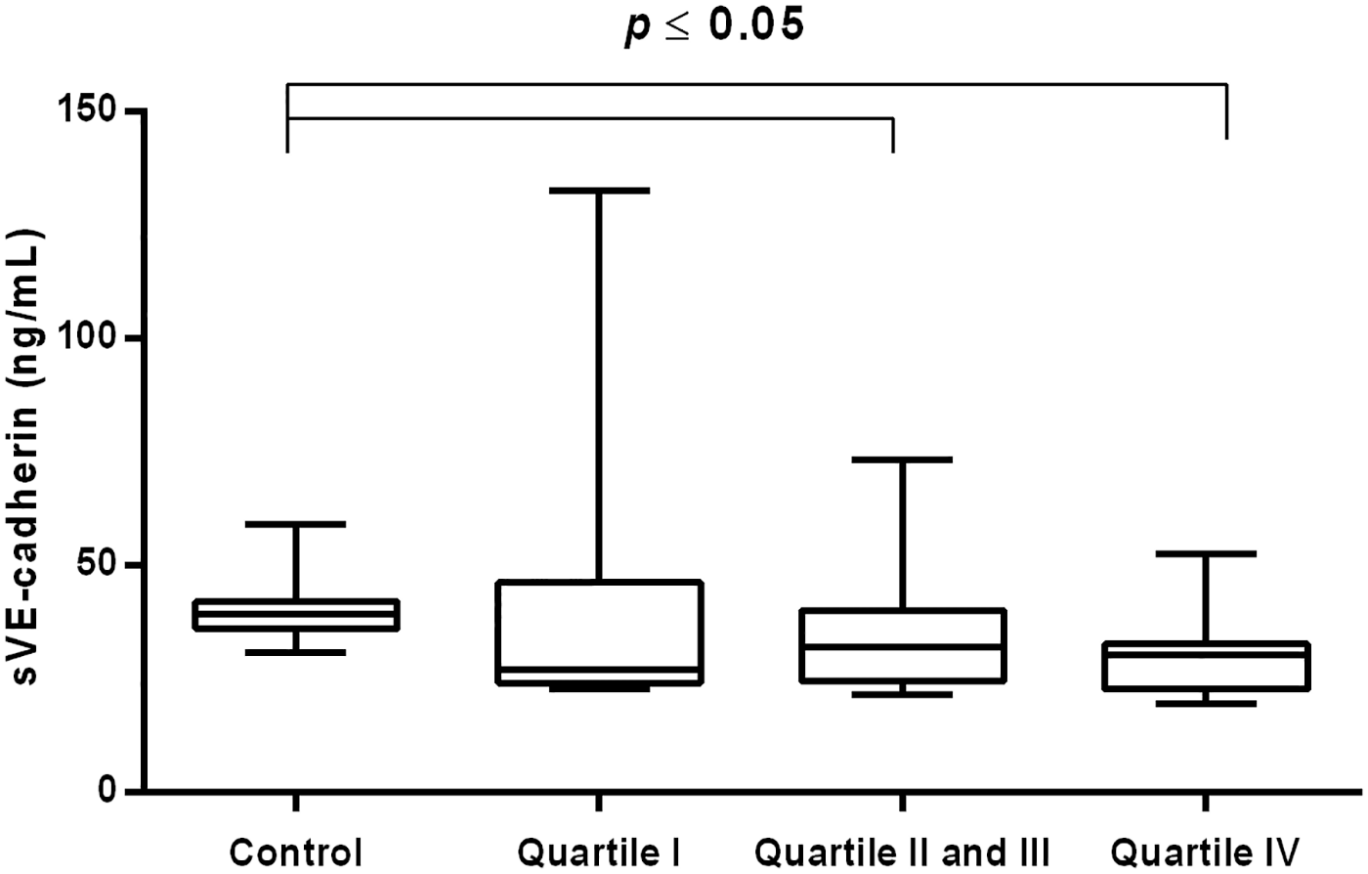
Concentration of sVE-cadherin in CVI woman categorized into quartiles of plasma elastase. Box and whisker plots show median (central line), upper and lower quartiles (box) and range excluding outliers (whiskers). Data were analyzed using Kruskal-Wallis test followed by the Dunn`s multiple comparison test. * *p*≤0.05 was considered statistically significant.

The concentration of sVE cadherin was decreased in CVI women in the middle and highest quartiles of hsCRP [32.29 (23.36–36.14) ng/mL vs. 39.30 (36.19–42.11) ng/mL *P* = 0.002; 28.51 (27.41–47.44) ng/mL vs. 39.30 (36.19–42.11) ng/mL *P* = 0.043] and elastase concentration [32.23 (24.60–40.18) ng/mL vs. 39.30 (36.19–42.11) ng/mL *P* = 0.022; 30.26 (22.83–32.77) ng/mL vs. 39.30 (36.19–42.11) ng/mL *P*<0.001], respectively (Figs 6 and 7).

**Fig 8:**
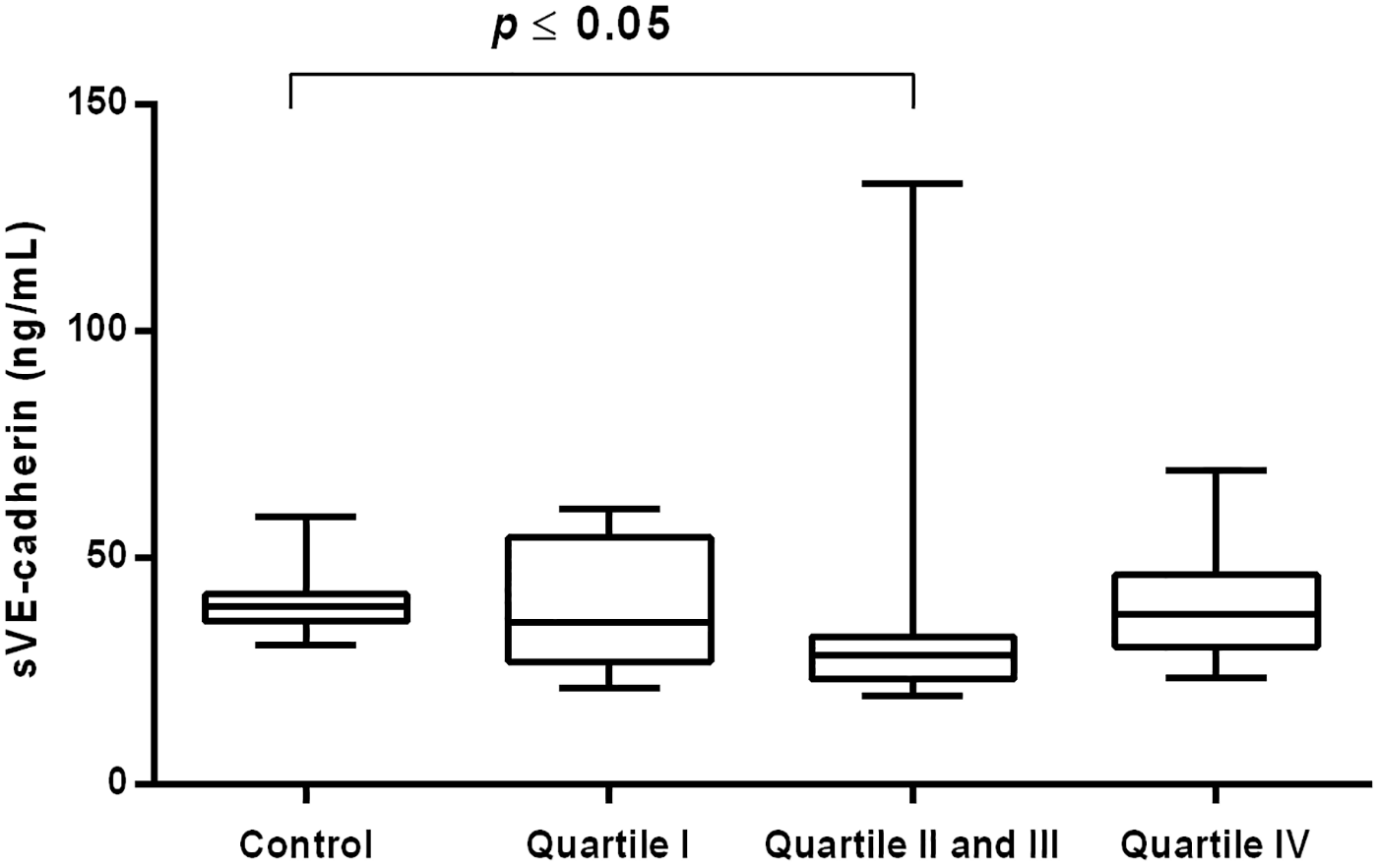
Concentration of sVE-cadherin in CVI woman categorized into quartiles of plasma MDA. Box and whisker plots show median (central line), upper and lower quartiles (box) and range excluding outliers (whiskers). Data were analyzed using Kruskal-Wallis test followed by the Dunn`s multiple comparison test. * *p*≤0.05 was considered statistically significant.

A significant fall in sVE-cadherin concentration was also noticed in the group of CVI women with the middle values of MDA [28.51 (23.32–32.69) ng/mL vs 39.30 (36.19–42.11) ng/mL *P*<0.0001] (Fig 8).

## Discussion

Numerous mechanisms have been proposed as the eatiology of CVI. One common hallmark of this disease is an elevated venous pressure and shift in the fluid shear stress [32]. These two conditions probably generate an abnormal biomechanical environment in the vein wall, which may initiate early activation of inflammatory cascade. Therefore, CVI is considered as a blood pressure-driven inflammatory disease, where inflammatory factors play a significant role [32]. Inflammation, accompanied by leukocyte activation and liberation of various cytokines, proteases, and reactive oxygen species (ROS) may affect morphology and function of endothelium of venules. The disruption of endothelial layer leads to the disturbance in specific vascular homeostasis, vascular tone, and permeability, resulting in the venous wall remodeling seen in all stages of CVI. The endothelial dysfunction is reflected either by the liberation of some specific molecules derived from damaged endothelial cells or the disruption in the concentration of factors naturally released by endothelium in physiological conditions. There is a lot of evidence which highlights the contribution of ED to the clinical status in cardiovascular diseases and shows a great potential of endothelial markers evaluation as a non-invasive method for predicting the disease progression and prognosis in patients with vascular disorders [33,34,35,36] Although ED is considered to be implicated in the pathogenesis of CVI, the number of studies assessing the endothelial status in patients with venous disease is still limited [28,29,30,31,37]. Little is known about the factors contributing to ED in CVI pathology, relationship of ED with the disease severity and the possible utility of endothelial markers in CVI diagnosis and monitoring. Due to the fact that CVI appears more commonly in women than in men [3,4,5,6], women seem to be an adequate model group for the investigation into the mechanisms and factors responsible for the disease occurrence and progression. Therefore the aim of the present study was to evaluate endothelial status in CVI women by measuring selected endothelial markers. The second purpose was to analyze the association of markers of ED with disease severity and factors potentially contributing to endothelial injury such as inflammation and oxidative stress.

In our study we chose four markers of endothelial dysfunction: vWf, sP-selectin, sTM and sVE-cadherin. The first marker mentioned, namely vWf, is a large glycoprotein that is required for normal hemostasis. It mediates in platelet aggregation and adhesion to the vascular wall, serves as a plasma carrier for factor VIII. vWf is produced and stored in Weibel-Palade bodies of endothelial cells. An increased liberation of vWf to circulation is followed by endothelial damage [38]. The elevated concentration of vWf has been found in diseases such as atherosclerosis, diabetes, rheumatoid vasculitis where damage to the endothelium is observed [39,40].

P-selectin is a cell adhesion molecule found alike vWf in the Weibel-Palade bodies of ECs. It can be rapidly released from the Weibal-Palade bodies of endothelial cells after appropriate activation by histamine, thrombin, complement, or ROS and mediate in leukocyte “rolling” on endothelium [41,42]. It has been suggested that plasma sP-selectin may reflect the functional status of ECs [43]. Its significant rise was demonstrated in stroke, acute myocardial infarction, coronary artery disease, and peripheral artery disease [44].

TM is one of the most popular indicators of endothelial injury, located on the vascular endothelium surface which functions as an anticoagulant. TM has an affinity for thrombin, forming a 1:1 thrombin-thrombomodulin complex that inhibits fibrin formation, platelet activation and protein S inactivation [45]. Apart from the transmembrane form, TM also occurs in soluble forms (sTM) in the plasma, which is probably the product of the cleaved transmembrane glycoprotein [46]. *In vitro* studies demonstrate that sTM is released from endothelial cells following cell membrane injury caused by the action of neutrophil derived proteases and oxygen radicals [47,48,49].

VE-cadherin is a novel marker of endothelial damage with a high specificity [50,51]. Contrary to the previously described molecules which could be liberated by other cell types, VE-cadherin is specifically expressed by endothelial cells [52]. VE-cadherin belongs to a large family of endothelial cadherin proteins. As a transmembrane molecule, it is located in the intracellular junctions where it regulates the barrier function of endothelium. Soluble VE-cadherin (sVE-cadherin) can be released into the blood after increased proteolytic activity mediated by metalloproteinases and other proteases [53]. Although sVE-cadherin has been evaluated in diseases associated with vascular endothelial injury [54,55,56] our study is the first to investigate the concentration of this protein in CVI.

Our study demonstrates a high concentration of sTM in CVI women which shows a significant trend to rise together with disease severity. This result suggests that the gradation of CVI severity might be connected with endothelial damage expansion. Moreover, we also observed that severe CVI is correlated with ageing and BMI value. These findings are in line with the results of Musil et al. who showed that age and BMI are significant predictors of clinical grade of venous disease according to the CEAP classification [57]. The authors observed that a greater age and elevated BMI were associated with an increased CEAP grade of visible disease and an increased risk of the clinical progression from varicose veins to trophic skin changes or venous ulcers. However, the predictive value of BMI was demonstrated only in women, which may suggest the existence of gender-specific factors predisposing to CVI development and progression. Due to the fact that increasing age and a high BMI value have been recognized as the factors contributing to ED [58,59,60], their influence on the endothelial abnormalities in CVI should also be taken into consideration.

Since it is known that sVE-cadherin may reflect endothelial barrier disruption we had expected to obtain an increased level of this molecule in our patients highly prone to endothelial injury. Surprisingly, both in moderate and severe CVI a decreased concentration of sVE-cadherin was observed. Although the rise of sVE-cadherin was noticed in patients with various vascular complications, there are some studies presenting results similar to ours. A decreased concentration of sVE-cadherin was demonstrated in patients infected by Shiga toxin 2 producing *E*. *coli* strain (STEC) and with the haemolytic ureamic syndrome (HUS), a complication caused by this bacteria and occurs via endothelial cell damage [61]. The study by Ebihara et al. shows that sepsis, a pathology accompanied by massive endothelial disruption and damage, is associated with decreased sVE-cadherin level [62]. Ostrowski et al., who investigated the markers of endothelial damage in patients with severe sepsis, obtained results identical to ours, namely a fall in sVE-cadherin concentration accompanied by the rise in sTM level [63]. One explanation of a decreased circulating level of sVE-cadherin suggested by the authors is an enhanced sequestration of this protein from the endothelial cell surface which may be induced by inflammatory mediators and ROS [64,65]. Sequestration of junctional VE-cadherin may decrease adhesive bonds between apposed endothelial cells and increase endothelial permeability. Therefore, a low concentration of circulating VE-cadherin presents either in moderate or severe stage of CVI may reflect a perturbation in endothelial barrier that occurs already at the initial stage of venous disease development.

In our study, there was no change in sWf and sP-selectin concentration at any stage of CVI severity. Our results are in contrast to the findings by Yasim et al. who observed an elevated concentration of vWf in patients with primary varicoce veins [66]. However, the authors included lower number of patients classified as C2 according to CEAP system. Bryan et al. revealed that a higher circulating sP-selectin is associated only with severe CVI and not with CVI overall [67]. However the correlation of sP-selectin and vWf with elastase and hsCRP, respectively, observed in the present study, suggests that they may be indicators of inflammation rather than ED. It is well-known that vWf is an acute phase reactant affected by inflammatory cytokines and as such, may be elevated even in the absence of ED [32]. The correlation of sP-selectin with platelet count demonstrated in previous studies [68,69] proves the utility of measuring this molecule as an inflammatory marker as well. sTM and sVE-cadherin seem to be less influenced by inflammation, and their concentrations remained constant regardless of hsCRP and elastase levels. This observation confirm a well-known fact that sTM, contrary to others endothelial markers, does not increase in an acute response to a variety of biological stimulations and, therefore, is the most likely a specific a marker of endothelial lesions and not of other cell types activation [45]. The sVE-cadherin seems to exhibit similar resistance to inflammatory stimuli. These findings suggest that these two particles may be markers of the true ED and that the concentration thereof, is dependent only on the actual endothelial status. Moreover, due to the fact that both sTM and sVE-cadherin already occur at an early stage of CVI, they may act as early markers of ED.

In our study the markers of inflammation and oxidative stress were used as the criteria to divide CVI women into appropriate groups with low, medium, and high inflammatory or oxidative stress status, respectively, in which endothelial parameters were evaluated and compared with healthy controls. This approach allowed to state which group of CVI women is particularly prone to endothelial injury. CVI women with the highest plasma hsCRP or elastase concentration, respectively, demonstrated an elevated value of sTM, which points to the inflammation as an important factor contributing to endothelial damage. A large body of evidence shows an increased level of oxidative stress parameters in the blood and varicose vein wall of CVI patients [70,71,72,73]. These findings were also confirmed by our study in which an elevated concentration of MDA was observed in plasma of CVI women. ROS liberated by inflammatory cells may have a direct effects on endothelial layer. Some experiments with monolayers of cultured endothelium demonstrated that ROS induce the cytolysis of endothelial cells and disruptions in endothelial cell adhesion [74,75]. Therefore, a simultaneous rise of sTM and MDA demonstrated in the present study may be the result of disruptive effects of enhanced oxidative stress on the endothelial layer, which escalate together with CVI severity. The contribution of ROS to endothelial perturbation is also confirmed by an increased concentration of sTM observed especially in CVI women with highest oxidative status. Moreover, the unique behavior of sVE-cadherin which starts to decrease in CVI women with the moderate plasma level of hsCRP, elastase and MDA respectively, suggests that not necessarily intense but even a mild inflammation or/and oxidative stress process may negatively influenced the endothelial barrier by decreasing the content of protein in endothelial junctions and increasing endothelial permeability.

We are aware of the limitation of this pilot study. First, it can be agreed that the number of CVI female participants was small However, the present work calls attention to the important aspect of biochemical markers of ED in CVI prognosis or/and diagnosis that has not yet been evaluated. These preliminary observations are therefore a potential concept for future clinical studies involving a larger cohort of patients. Second, the use of MDA as an oxidative stress marker may be also controversial. Although MDA is a widely accepted assay for oxidative damage, the most common methods of MDA detection, TBA test, shows several pitfalls and has been criticized as being too unspecific and prone to artifacts. TBA can react with several compounds, including sugars, amino acids, bilirubin, and albumin, producing interferences in the measurement. MDA assay can be used in association with other indices of lipid peroxidation, such as 4-hydroxynnenal, conjugated dienes, ethane and pentane gases, and isoprostanes. Unluckily, these methods have limitations because they are either too expensive, too time consuming or their application needs specialized personnel [76]. Therefore one of the most important reason of choosing of MDA assay in the present study was its simplicity, rapidity and possibility of implementation in routine clinical testing.

## Conclusions

The results of the present study demonstrates the presence of ED in women suffering from CVI which progresses with the disease severity and may be associated with inflammation and enhanced oxidative stress. Due to the fact that both sTM and sVE-cadherin already occur at an early stage of CVI, they may act as early markers of ED. Moreover, our findings, assumedly for the first time, demonstrate that endothelial perturbation may involve an increased endothelial permeability that can occur even at an early stage of CVI development.

## Supporting information

S1 Fig Correlation between sP-selectin and elastase. (Spearman correlation coefficient r = 0.419, *P* = 0.014).
(PDF)

S2 Fig Correlation between vWF and hsCRP. (Spearman correlation coefficient r = 0.373, *P* = 0.049).
(PDF)

S3 Fig Concentration of sTM in CVI woman categorized into quartiles of plasma hsCRP. Box and whisker plots show median (central line), upper and lower quartiles (box) and range excluding outliers (whiskers). Data were analyzed using Kruskal-Wallis test followed by the Dunn`s multiple comparison test. * *P*≤0.05 was considered statistically significant.
(PDF)

S4 Fig Concentration of sTM in CVI woman categorized into quartiles of plasma elastase. Box and whisker plots show median (central line), upper and lower quartiles (box) and range excluding outliers (whiskers). Data were analyzed using Kruskal-Wallis test followed by the Dunn`s multiple comparison test. * *p*≤0.05 was considered statistically significant.
(PDF)

S5 Fig Concentration of sTM in CVI woman categorized into quartiles of plasma MDA. Box and whisker plots show median (central line), upper and lower quartiles (box) and range excluding outliers (whiskers). Data were analyzed using Kruskal-Wallis test followed by the Dunn`s multiple comparison test. * *p*≤0.05 was considered statistically significant.
(PDF)

S6 Fig Concentration of sVE-cadherin in CVI woman categorized into quartiles of plasma hsCRP and control group. Box and whisker plots show median (central line), upper and lower quartiles (box) and range excluding outliers (whiskers). Data were analyzed using Kruskal-Wallis test followed by the Dunn`s multiple comparison test. * *p*≤0.05 was considered statistically significant.
(PDF)

S7 Fig Concentration of sVE-cadherin in CVI woman categorized into quartiles of plasma elastase. Box and whisker plots show median (central line), upper and lower quartiles (box) and range excluding outliers (whiskers). Data were analyzed using Kruskal-Wallis test followed by the Dunn`s multiple comparison test. * *p*≤0.05 was considered statistically significant.
(PDF)

S8 Fig Concentration of sVE-cadherin in CVI woman categorized into quartiles of plasma MDA. Box and whisker plots show median (central line), upper and lower quartiles (box) and range excluding outliers (whiskers). Data were analyzed using Kruskal-Wallis test followed by the Dunn`s multiple comparison test. * *p*≤0.05 was considered statistically significant.
(PDF)

S1 Table Markers of endothelial dysfunction, inflammation and oxidative stress in women with moderate and severe CVI. ^(a)^Results shown as mean± standard deviation, one-way ANOVA test was used for comparison.^(b)^Results shown as median and interquartile range, Kruskull-Wallis test was used for comparison.NS—not statistically significant.
(PDF)
