# Supplementary figures and images for: Plasma concentration of selected biochemical markers of endothelial dysfunction in women with various severity of chronic venous insufficiency (CVI)—A pilot study
Source: PLoS One. 2018 Jan 29;13(1):e0191902. doi: 10.1371/journal.pone.0191902 (PMC5788369; doi:10.1371/journal.pone.0191902)

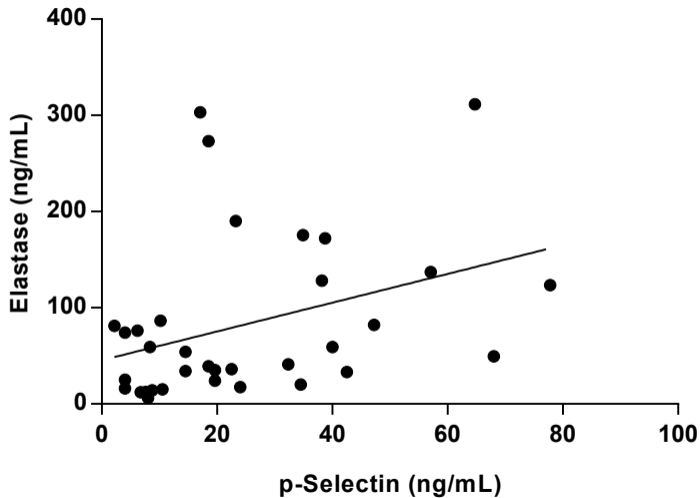

Supplement: S1 Fig — (Spearman correlation coefficient r = 0.419, P = 0.014). (PDF) [file pone.0191902.s001.pdf]

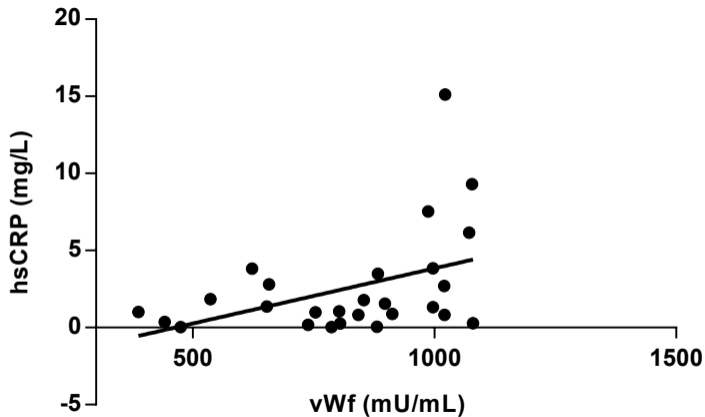

Supplement: S2 Fig — (Spearman correlation coefficient r = 0.373, P = 0.049). (PDF) [file pone.0191902.s002.pdf]

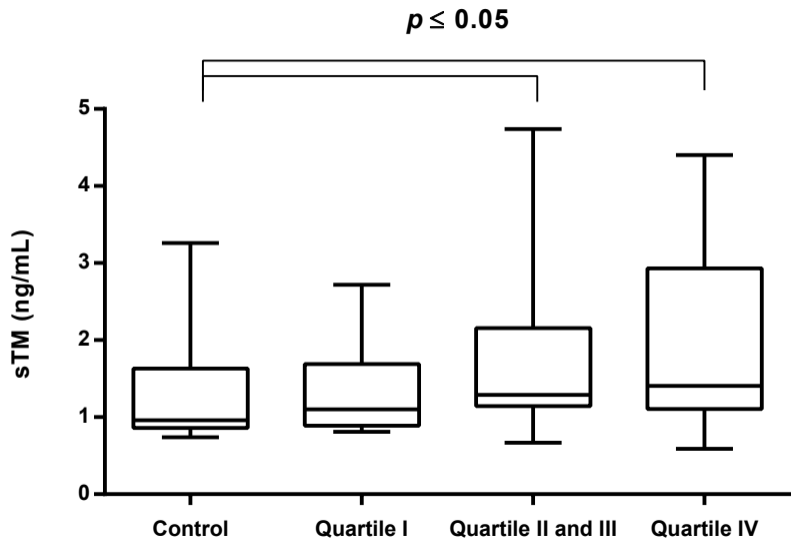

Supplement: S3 Fig — Box and whisker plots show median (central line), upper and lower quartiles (box) and range excluding outliers (whiskers). Data were analyzed using Kruskal-Wallis test followed by the Dunn`s multiple comparison test. * P≤0.05 was considered statistically significant. (PDF) [file pone.0191902.s003.pdf]

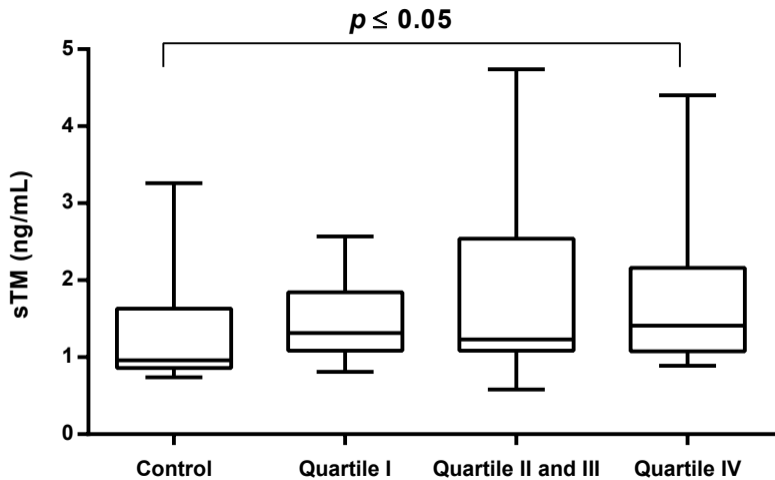

Supplement: S4 Fig — Box and whisker plots show median (central line), upper and lower quartiles (box) and range excluding outliers (whiskers). Data were analyzed using Kruskal-Wallis test followed by the Dunn`s multiple comparison test. * p≤0.05 was considered statistically significant. (PDF) [file pone.0191902.s004.pdf]

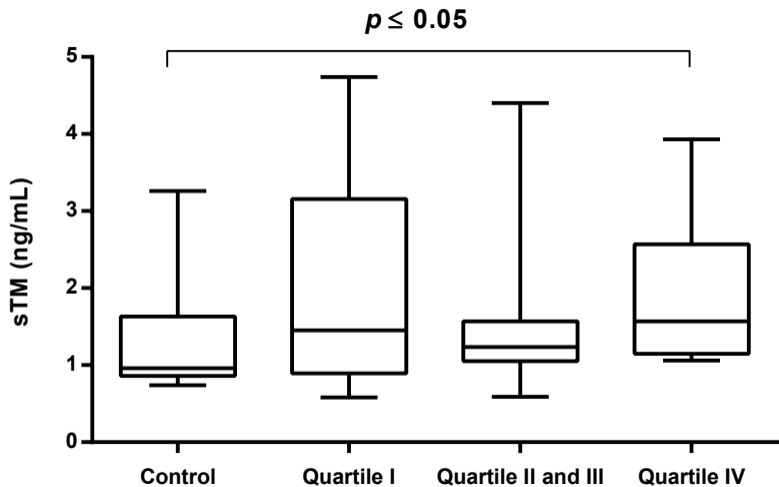

Supplement: S5 Fig — Box and whisker plots show median (central line), upper and lower quartiles (box) and range excluding outliers (whiskers). Data were analyzed using Kruskal-Wallis test followed by the Dunn`s multiple comparison test. * p≤0.05 was considered statistically significant. (PDF) [file pone.0191902.s005.pdf]

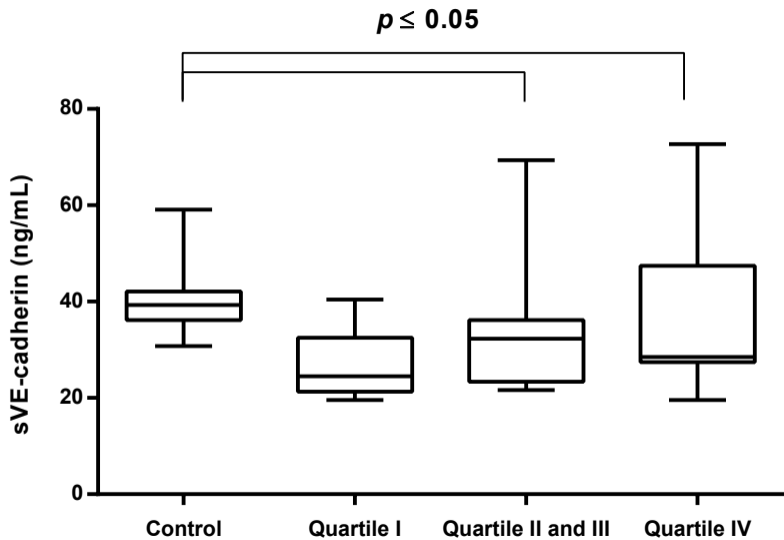

Supplement: S6 Fig — Box and whisker plots show median (central line), upper and lower quartiles (box) and range excluding outliers (whiskers). Data were analyzed using Kruskal-Wallis test followed by the Dunn`s multiple comparison test. * p≤0.05 was considered statistically significant. (PDF) [file pone.0191902.s006.pdf]

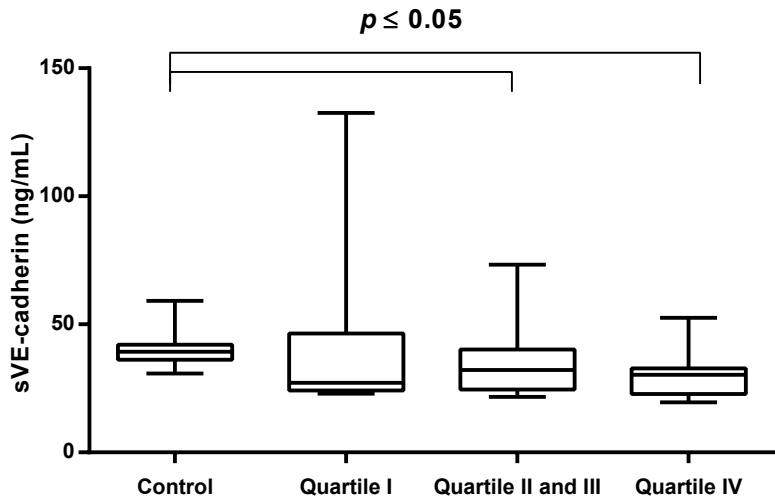

Supplement: S7 Fig — Box and whisker plots show median (central line), upper and lower quartiles (box) and range excluding outliers (whiskers). Data were analyzed using Kruskal-Wallis test followed by the Dunn`s multiple comparison test. * p≤0.05 was considered statistically significant. (PDF) [file pone.0191902.s007.pdf]

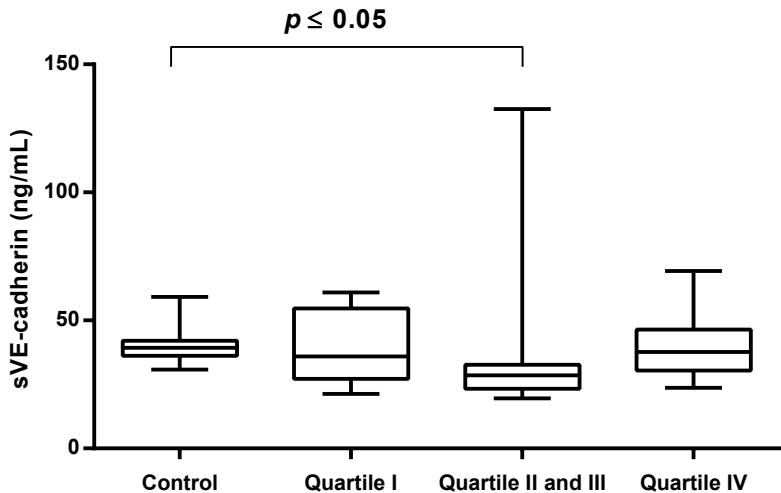

Supplement: S8 Fig — Box and whisker plots show median (central line), upper and lower quartiles (box) and range excluding outliers (whiskers). Data were analyzed using Kruskal-Wallis test followed by the Dunn`s multiple comparison test. * p≤0.05 was considered statistically significant. (PDF) [file pone.0191902.s008.pdf]
