# Supplementary material for: Plasma concentration of selected biochemical markers of endothelial dysfunction in women with various severity of chronic venous insufficiency (CVI)—A pilot study
Source: PLoS One. 2018 Jan 29;13(1):e0191902. doi: 10.1371/journal.pone.0191902 (PMC5788369; doi:10.1371/journal.pone.0191902)
Supplement: S1 Table — (a)Results shown as mean± standard deviation, one-way ANOVA test was used for comparison.(b)Results shown as median and interquartile range, Kruskull-Wallis test was used for comparison.NS—not statistically significant. (PDF) [file pone.0191902.s009.pdf]

**Parameter**	**Control (*n*=33)**	**Moderate CVI (*n*=28)**	**Severe CVI (*n*=16)**	***p*-value**
Age (years)	45.18±10.53	41.72±9.15	51.44±10.22	0.001[(a)]
BMI (kg/m^2)	24.52±6.34	22.67±4.83	26.19±5.06	0.027[(a)]
hsCRP (mg/L)	1.00 (0.12-1.86)	0.98 (0.13-3.15)	1.15 (0.56-2.84)	NS[(b)]
Elastase (ng/mL)	41.50 (24.50-65.75)	51.75 (19.00-81.25)	59.00 (25-172)	NS[(b)]
MDA (µM)	2.41 (2.03-4.06)	3.93 (2.92-5.40)	4.41 (3.47-5.93)	0.001[(b)]
vWF (mU/mL)	808 (723-957)	890 (649-961)	805 (627-997)	NS[(b)]
sTM (ng/mL)	0.96 (0.86-1.63)	1.23 (1.09-1.69)	1.43 (1.10-2.25)	0.027[(b)]
P-selectin (ng/mL)	26.16 (11.45-45.82)	19.64 (8-36.36)	21.27 (10.27-52.50)	NS[(b)]
sVE-cadherin (ng/mL)	39.30 (36.19-42.11)	30.34 (23.36-34.44)	31.56 (26.31-41.90)	0.002[(b)]
